# Geopolymer, Calcium Aluminate, and Portland Cement-Based Mortars: Comparing Degradation Using Acetic Acid

**DOI:** 10.3390/ma12193115

**Published:** 2019-09-24

**Authors:** Neven Ukrainczyk, Murugan Muthu, Oliver Vogt, Eddie Koenders

**Affiliations:** 1 Institute of Construction and Building Materials, TU Darmstadt, 64287 Darmstadt, Germany; vogt@wib.tu-darmstadt.de (O.V.); koenders@wib.tu-darmstadt.de (E.K.); 2 School of Civil and Environmental Engineering, Nanyang Technological University, 50 Nanyang Avenue, Singapore 639798, Singapore; murugan88m@gmail.com

**Keywords:** geopolymer, Portland cement, calcium aluminate cement, leaching, microstructure, deterioration, acetic acid attack, SEM-EDS

## Abstract

In this paper, we comparitvley studied acetic acid attacks on geopolymer (GP-M), calcium aluminate (CAC-M), and Portland cement (PC-M)-based mortars. Consequent formations of deteriorated or transition layers surrounding the unaltered core material was classified in these three mortars, according to different degradation levels depending on what binder type was involved. Apart from mass loss, hardness, and deterioration depth, their microstructural alterations were analyzed using test methods such as scanning electron microscopy with energy dispersive spectroscopy (SEM-EDS), mercury intrusion porosimetry (MIP), powder X-ray diffraction (XRD), and thermogravimetric analysis-differential scanning calorimeter (TGA-DSC), which showed the different mechanisms for each binder type. Elemental maps revealed the decalcification (PC-M and CAC-M) and depolymerization (GP-M) that occurred across the mortar sections. The mass loss, hardness, and porosity were the least affected for GP-M, followed by CAC-M. These results points out that geopolymer-based mortars have improved acid resistance, which can be used as a potential alternative to conventional cement concretes that have been exposed to agro-industrial environments.

## 1. Introduction

Reinforced concrete structures frequently exposed to various organic acids (i.e., acetic, citric, lactic, propionic, and butyric) operate in wine, animal rearing, sugar, and dairy industries. The aggressiveness of such aqueous media is dependent on the acid’s constant dissociation (propionic > butyric > acetic > lactic), indicating the solubility degree of acid salts in water [1]. This acid attack deteriorates the binder involved, which seemingly reduces the service life of concrete [2,3]. Among the different acids from agro-industrial waste waters, an acetic acid attack on cement concrete is the most common type. It progressively dissolves the binder phases, wherein carbonate aggregates follow a diffusion-dissolution-precipitation controlled deterioration process. Meanwhile, the siliceous aggregates remain chemically unaltered [4]. 

Portland cement (PC) is the main construction material used worldwide to build concrete structures. The protons from acid species diffusing into the PC-based concrete first dissolves the portlandite (C–H) phase along with monosulphate (AFm) and ettringite (AFt), and thereafter decalcifies calcium-silicate-hydrate (C–S–H) and unhydrated cement. As a consequence, the acetate ions form weak complexes with Ca, Al, and Fe(II) ions. Nevertheless, it has stronger complexes with Fe(III) [5]. Strongly deteriorated concrete materials exposed to acids, regardless of acid type, are often characterized by a light-brown discoloration at the deterioration front, which is the precipitation of ferric hydroxide. This kind of chemical imbalance alters the concrete microstructure, which apparently reduces its mechanical strength and may lead to reinforcement corrosion [4].

It was attempted several times to improve the acid resistance of concrete by strengthening its microstructure. On one hand, ordinary Portland cement (OPC) is improved by using supplementary cementing materials (SCMs) like fly ash, silica fume, slag, and metakaolin [6,7]. SCMs refine the pore-structure and further stabilize the chemical structure—i.e., convert portlandite into C–S–H—which therefore reduces the diffusion of acid species from external solution. On the other hand, the acid resistance of concrete systems prepared using alternative binders like conventional calcium aluminate cement (CAC) and novel alkali activated materials, i.e. geopolymers (GP), have a promising performance [8]. The CACs commonly applied in real conditions demand high early strength, low ambient temperature placement, refractory, and high resistance to chemical attacks and abrasion [9,10]. Unlike PC, the CAC has a lower Ca leaching rate (due to the absence of portlandite), a lower Ca concentration, and a different pH (neutralization) buffering behavior [11]. While aiming to cut back on cement’s carbon footprint, the construction industry is slowly transforming towards a cement free era, where GP-based concrete could be a potential substitute, especially for niche applications. GPs are mineral binders that exist when mixing an alkali activator with a reactive aluminosilicate powder like fly ash, slag, calcined clay, or other pozzolans [12,13]. Metakaolin (MK), a type of calcined clay, is preferred as a precursor because of its high reactivity. Fly ash, on the other hand, requires heat curing. GP binders are characterized by negligible calcium content, unlike PC, CAC, and alkali activated-based ones (e.g., slag), and therefore exhibit improved resistance to aggressive acidic solutions [14,15].

Understanding the effect of different binders on the deterioration characteristics of concrete is of primary interest in this study. Thus, it suggests that the modifications required to improve the concrete performance in acidic environment. The present study demonstrates a 44-day acetic acid attack on GP, CAC, and PC-based mortars. Thereafter, we measured their mass loss, hardness, and deterioration depths. Microstructural alterations were analyzed in depth using test methods like scanning electron microscopy with energy dispersive spectroscopy (SEM-EDS), mercury intrusion porosimetry (MIP), powder X-ray diffraction (XRD), and thermogravimetric analysis (TGA). On their polished sections, elemental maps were recorded to reveal the decalcification (PC- and CAC-based mortars) and depolymerization (GP mortar), which occurred across the mortar sections due to acid attacks. The overall findings aid in the understanding of chemical changes happening in the mortars, many of which use completely different binder types when exposed to an organic acid environment.

## 2. Materials and Methods 

### 2.1. Materials and Specimen Preparation

This study investigated three different mortar (-M) types made of geopolymer (GP), calcium aluminate cement (CAC), and Portland cement (PC). Herein, they were designated as PC-M, CAC-M, and GP-M, respectively. Holcim CEM I 52.5R Portland cement, Calucem ISTRA-50 calcium aluminate cement, metakaolin, and quartz sand (QS) that conformed to standards DIN 197-1, DIN 14647, and DIN 196-1 were used in the mortar preparations, respectively. Table 1 shows the properties of the raw materials used in this study. Tap water was used to prepare PC-M and CAC-M, but the GP-M was polymerized by mixing metakaolin (MK) and Geosil (GS) 14515 (a potassium-based silicate solution procured from Wöllner Germany) together. The workability (flow) spread and compression strength tests were conducted by conforming to DIN 1015-3 and DIN 1015-11 (using six samples). The mix design of the three mortars were chosen by fixing the workability spread at around 17 cm. The sand-to-binder ratio was fixed to 3 and the water-to-binder ratio was 0.5 for PC-M and 0.45 for CAC-M—the values were slightly different in order to achieve the same workability (and total porosity). The metakaolin-to-waterglass ratio of 0.94 was used for GP-M. This resulted in a higher total porosity for geopolymer samples (17%) than PC-M and CAC-M (both 15%), as measured by the water absorption method (on six 4 cm^3^ samples). The prismatic mortars (of standard size, 4 x 4 x 16 cm) were demolded after 24 h and then cured in tap water for 28 days. To avoid metastable hydration products, CAC-M was additionaly cured for one day at 60 °C. The compressive strength results showed a rapid strength increment for geopolymers, reaching 69 and 79 MPa in one and seven days, respectively. PC-M and CAC-M, meanwhile, reached only 38 and 50 MPa after seven days. 

Multiple (33) cut sections with nominal dimensions (11 mm thick, 20 mm width, and 40 mm long) were cut from the standard prisms using a diamond-tipped saw. These cut sections were then dried at 105 °C for 24 h and fully soaked in tap water for the next 24 h. Thereafter, their saturated mass was measured using a weigh balance with an accuracy of 1 mg.

### 2.2. Exposure to Acetic Acid

An acetic acid attack was performed by immersing the aforementioned saturated cut sections (33 replicates from each mix) in an acetic acid solution of 1.0 molar concentration (pH = 2) for 22 days and after a subsequent solution replenishment for another 22 days (44 days in total). The pH change in this solution with time was measured using a pH meter (Hanna pH 211, as an average of two replicate setups). The initial high acid concentration chosen and acid replenishement was adopted for this study to achieve quick results by accelerating the deterioration process, which was not representative to the real-field application. Then, the mass change in mortars after acid exposure for 22 and 44 days were measured, respectively. At these time periods, the deteriorated mortars were stored in acetone before the different characterization studies. In this paper, sample notations *t* + 0, *t* + 22, and *t* + 44 (time periods) indicate a cured (non-deteriorated) prism. Later, this prism was subjected to a 22 and 44 day acid attack. 

### 2.3. Characterisation Methods

Cross-section analyses for SEM and indentation (hardness) tests were performed only on *t* + 44 samples. The cut sections were impregnated with epoxy (low viscosity liquid) using a vacuum impregnation device. To reveal the sample surface, the hardened epoxy polymer (coarse) was polished at a rotational speed of 300 rpm using a resin bonded diamond disc (hardness range HV 150 to 2000) mounted on the grinding-polishing machine. It was again polished on a lubricated cloth mounted on a polishing machine to the desired level using an automated polycrystalline diamond spray of 9 µm, 3 µm, and 1 µm size at a rotational speed of 150 rpm. In the case of SEM (Zeiss EVO LS25, Jena, Germany ), the polished section of the deteriorated mortars were prepared to collect backscattered electrons (BSE) and EDS images (EDAX, AMETEK, Berwyn, USA). The light microscopy (Keyence VHX-600, Osaka, Japan) images were taken to inspect the deterioration depth across the mortar sections. Indentation (microhardness) tests were performed on an epoxy impregnated and polished samples using about 20 indents with Vickers geometry and 1 N loading (Fischerscope Hardness tester H100, Windsor, USA). Indentations were meticulously (manually) positioned using optical microscopy equipped with this device to indent the only binder matrix (degradative and unaltered layers) and avoid sand aggregates.

**Figure 1:**
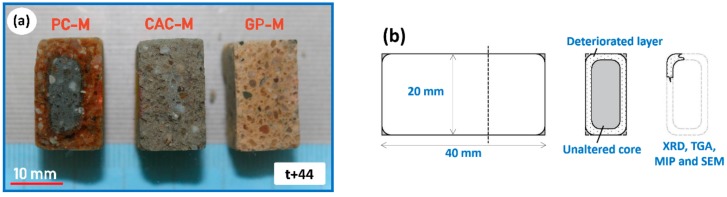
(**a**) Cut section revealing the deterioration across the three mortars. (**b**) Schematic illustrating the sample collection method. GP-M: geopolymer; CAC-M: calcium aluminate; PC-M: Portland cement; SEM: scanning electron microscopy; MIP: mercury intrusion porosimetry; XRD: X-ray diffraction; and TGA: thermogravimetric analysis.

The deteriorated layer noticed in the cut section of each *t* + 22 and *t* + 44 prismatic mortars (dried at 105 °C for 24 h) was carefully removed. The sample with a deteriorated layer and unaltered core (of equivalent volume) was carefully sectioned (Figure 1), dried (made acetone free), and then investigated with MIP (Thermo Scientific Pascal 140–440, Waltham, MA, USA). MIP measurements were performed up to 400 MPa (two replicate samples). The pore diameter was calculated using the Washburn equation following mercury properties: density 13.53 g/cm^3^, surface tension 0.485 N/m, and contact angle 130°. 

The sampled deteriorated layer (*t* + 0, *t* + 22, and *t* + 44), taken from two replicate specimens, was hand-milled and ground to a size less than 75 µm. An X-ray diffractometer was configured with CuKα_1,2_ radiation and a fast linear LYNXEYE detector (Bruker D2 PHASER, Hamburg, Germany). For the simultaneous thermogravimetry (TGA) with differential scanning calorimeter (DSC) study (Netzsch STA 449 F5, Selb, Germany), an alumina crucible filled with 50 mg of the sample was heated up to 1000 °C at a rate of 20 °C per min in an inert (N_2_) environment. To avoid the effect of remaining acetone, the sample was kept at an isothermal condition (40 °C) for 30 min during the test. Chemically bound water, Ca(OH)_2_, and CaCO_3_ were obtained from mass losses between about 40–600 °C, 400–500 °C, and 550–750 °C, respectively. The results were normalized to the (remaining) binder content, in which the sand content was eliminated, according to Sheffield [5], Weise et al. [6], and Ramachandran et al. [7]. A quartz α-β phase transition, seen in the DSC signal around 580 °C, was employed in order to quantify the quartz content in the sample, and therefore binder as well, based on the calibration with pure standard sand.

## 3. Results

Optical images (Figure 1a) clearly show how the acid attack on the three mortars resulted in the formation of different deteriorated and transition layers, which surrounded the unaltered core material. In PC-M, a white deteriorated layer and brown discoloration that surrounded the unaltered core was visually observed. A light-gray deteriorated layer surrounding the gray unaltered core were noticed in CAC-M. Much less color change was seen in GP-M. With light microscopy imaging (of the four cut sections from each mortar), the average thicknesses of the deteriorated layers in PC-M, CAC-M, and the transition layer in GP-M (named transition due to improved properties shown later) were obtained using the microscopy built-in image analyzing tools. They were found to be 3.7 mm, 4.8 mm, and 2.5 mm, respectively. Spraying phenolphthalein solution over the GP-M cut section resulted in a colorless effect, which indicated a pH less than 8.2. 

**Figure 2:**
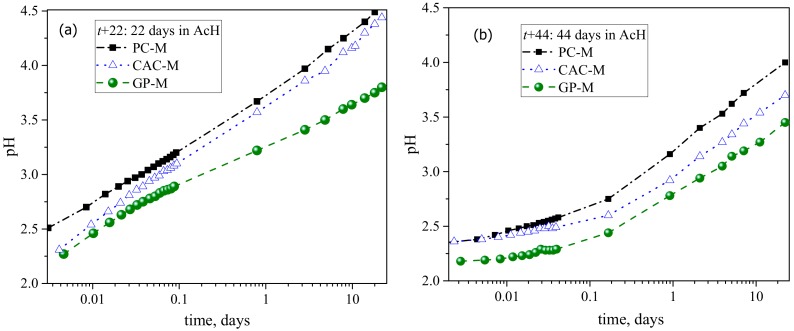
pH evolution of external solution, which indicates the rate of neutralization due to leaching/dissolution of the mortars: (**a**) before and (**b**) after the solution renewal.

The pH evolution of the external solution (Figure 2), shows that the neutralization rate followed the order PC-M > CAC-M > GP-M for both the initial exposure (*t* + 22 days) and after the acid solution replenishment (*t* + 44 days). Compared to the initial exposure (*t* + 22 days), the neutralization rate after the solution renewal was lower for all three mortar types (Figure 2a,b).

**Figure 3:**
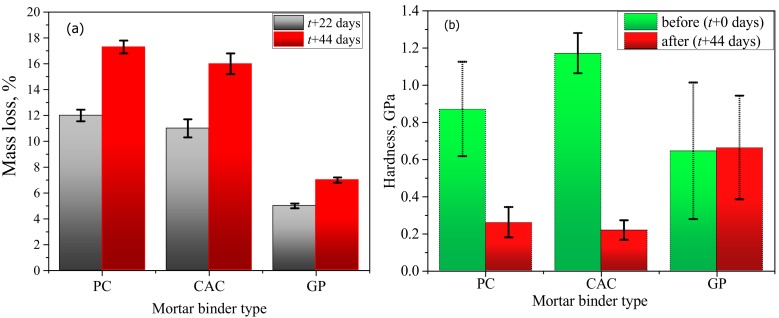
Effect of acetic acid on: (**a**) macroscopic mass loss and (**b**) microhardness (error bars represent ± one standard deviation).

The mass loss in PC-M, CAC-M, and GP-M after an acid exposure of 22 days was 12%, 11%, and 5%; after 44 days, it was 17%, 16% and 7% (Figure 3a). These results indicate that the deterioration degree in GP-M was significantly less for PC-M and CAC-M. Results of the hardness indentation in the binder matrix (Figure 3b) also confirmed that the reduction in the mechanical properties of the degradative/transition layer was most for CAC, followed by PC, and none for GP.

**Figure 4:**
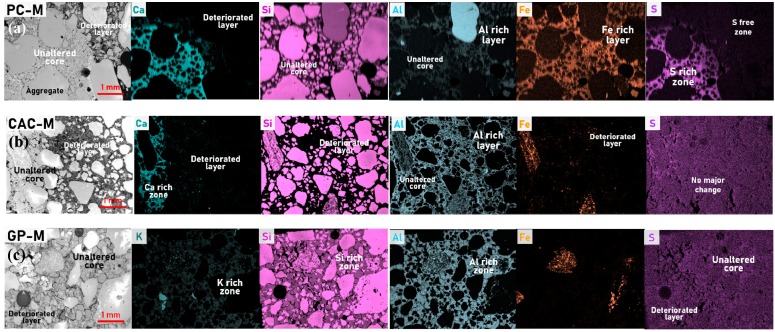
SEM-BSD micrographs (60x magnification) and corresponding energy dispersive spectroscopy (EDS) elemental (colored) maps revealing the phase change along the cross section of (**a**) PC-M, (**b**) CAC-M, and (**c**) GP-M subjected to a 44-day acid attack.

**Figure 5:**
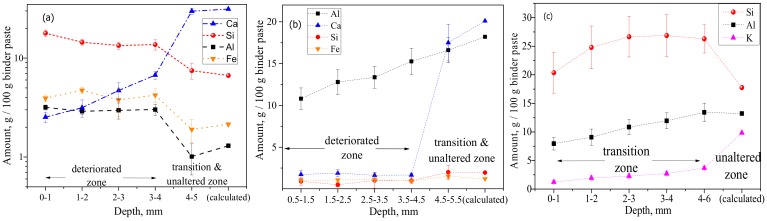
Chemical composition as a function of degradation depth, obtained from the statistical analysis of EDS mappings, which filtered out (disregarding pixels/areas related to) sand aggregates and only considered binder paste: (**a**) PC, (**b**) CAC, and (**c**) GP (error bars represent ± one standard deviation).

SEM-BSD (backscattered electrons detector) micrographs and high-resolution EDS elemental maps on cross-sections revealed the deterioration degree and phase changes in the three mortars after 44 days of exposure (Figure 4 and Figure 5). The deteriorated layer in PC-M and CAC-M appeared to be highly porous, soft, and weak (based on polishing morphology identified on SEM-SE images (not shown here) and hardness results; Figure 3b). GP-M, meanwhile, was less affected. The remaining main elements in the deteriorated layer of the three mortars were clearly revealed by their elemental maps (Figure 4). They were Si, Al, Fe, and C for PC-M; Al and C for CAC-M; and Si, Al, and C for GP-M. On the other hand, the major elements that seemed to leach out due to acid exposure on PC-M (Ca and S), CAC-M (Ca), and GP-M (K) were also clearly noticed in the EDS results. On the whole, the elemental maps confirmed the precipitation of silica gel (PC-M), alumina gel (CAC-M), and Si rich geopolymer (GP-M) in the deteriorated layer of the three mortars (Figure 4). The carbon presence indicates the epoxy, which corresponds to increased porosity.

Depth profiles of elemental composition (Figure 5) were obtained from statistical analysis of EDS mappings. Only the binder paste was considered by filtering out the sand aggregates. The mass percentage was expressed per 100 g of binder paste, where the binder paste included carbon (C) as an approximation for porosity. This was related to the epoxy impregnation, wherein the sum of the major (heavier) elements (i.e., Ca, Si, Al, O, S, Fe, and C) could roughly approximate a water saturated binder paste, where C^12^ was approximated for O^16^, and H^1^ was disregarded. Although considered only as semi-quantitative (not absolute), quantitative significance could be tempted when comparing the inner (unaltered) core material composition with the theoretical one, which could be calculated from the original mix design composition. For PC and CAC, good agreements were found for Ca and Si, whereas for GP, significantly higher amounts of measured Si could be attributed to dissolution of (quartz) sand aggregates and their increased incorporation in the GP gel matrix. (More research is needed here to confirm that.) The deterioration depths (Figure 5) followed the order CAC-M (4.5 mm) > PC-M (4 mm) > GP-M (2 mm) and agree well with the optical micrographs image analysis results. In CAC, the calcium (Ca) profile had a sharp jump (from 21% to 2%) around a depth of 4.5 mm. This value was considered as a deterioration depth, rich in alumina gel and practically absent of C_3_AH_6_ (confirmed using XRD, as shown later). The aluminum (Al) profile steadily decreased from an initial 18.2% (calculated for an unaltered core) to 10.8%. This profile was measured at the outmost surface layer, which indicates a possible gradual leaching (dissolution) of alumina gel. In PC, a sharp jump in calcium occurred around a depth of 4 mm, dropping from 30% to 6.7%. This indicates the disappearance of Ca(OH)_2_. From this threshold depth, a gradual decrease of Ca toward the outermost surface layer agreed with the further incongruity of C-S-H decalcification. Higher levels of Si, Al, and Fe were detected in the deteriorated layer (i.e., in the unaltered zone), which could be due to the formation of corresponding gels. In GP, the Si profile indicates its dissolution until reaching a depth of 2 mm, which might be considered a degradation zone. However, due to its low magnitude, lack of significant change in microhardness (indentation in binder matrix, Figure 3b), and porosity (MIP, Table 2), this can be considered as a transition zone. Moreover, this zone exhibits gradual dealumination (the decrease of Al content from 13% to 8%) and strong leaching of K^+^ due to ion exchange [8,9].

**Figure 6:**
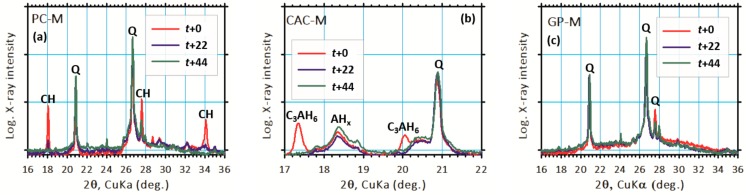
XRD results revealing major changes in binder phases due to the acid attack of (**a**) PC-M, (**b**) CAC-M, and (**c**) GP-M mortars.

The XRD peaks at 18.08° and 34.08° 2θ correspond to the portlandite presence in PC-M (Figure 6a), indicating a partial (22 days) and complete (44 days) disappearance with an increase in acid exposure time. In the case of CAC-M, the diffraction peaks at 17.34º and 20.06º 2θ (Figure 6b) indicate complete dissolution of the C_3_AH_6_ phase after 22 days. For GP-M, the acid attack had no major influence on the crystalline phases, which was clearly noticed in its XRD patterns. This, of course, indicated a quartz presence (Figure 6c). However, a distinct (GP) amorphous hump was observed shifting toward lower diffraction (2θ) angles. This result is opposite of a common qualitative analysis of geopolymerization [10], which is based on how far an amorphous hump shifts to higher diffraction (2θ) angles. Therefore, the observed shift might suggest the depolymerization of GPs. 

**Figure 7:**
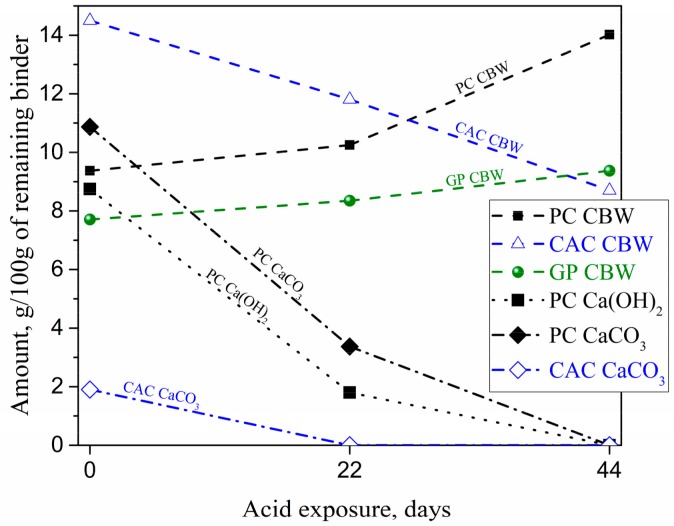
Changes in chemically bound water (CBW), Ca(OH)_2_, and CaCO_3_ in the deteriorated layer of mortars as a function of acid exposure, obtained using thermogravimetric analysis-differential scanning calorimeter (TGA-DSC).

STA (TGA-DSC) analyses (Figure 7) provided semi-quantitative information on the changes in binders due to the acid attack. Amounts of chemically bounded water (CBW), Ca(OH)_2_, and CaCO_3_ were obtained from mass losses as detailed in Section 2.3. It was found that the CBW contents in PC-M and GP-M had increased, while the CBW in CAC-M had decreased with an increase in acid exposure time. The increase in bound water (normalized to the binder content) can be explained both by a leaching of a binder in deteriorated zones and the relatively higher water contents in newly precipitated silica, iron, and alumina gels. The acid exposure reduced the portlandite (and CaCO_3_) in PC-M from 8.8% to 1.8% (and 10.9% to 3.4%); whereas CaCO_3_ in CAC-M completely dissolved after 22 days.

MIP data characterized the changes in pore structure for acid attacks. The average pore size, gel, capillary, and macro porosities in mortars were determined (Table 2) by following Aligizaki [11] classification of the pores—gel pores as less than 10 nm; capillary pores between 10 nm to 10 µm; and macro pores are bigger than 10 µm. The 44 day acid attack increased the total pore volume in PC-M and CAC-M up to 85% and 74%, respectively, but no major change occurred in the GP-M. This indicates that the microstructure of GP-M has better chemical resistance than the other two mortars against the aggressive ionic species. The content of macro pores (>10 µm) accounts for the open porosity in the binder phase and in the aggregate-binder interface. The increase in such quantity clearly indicates the coarsening of pore structure in the two cement-based mortar systems (PC-M and CAC-M) due to acid exposure. Compared to the macro pores, the capillary pores content (i.e., pores size less than 10 µm) in CAC-M was relatively low, probably because of the initially coarse size distribution induced by the well-documented conversion reactions (CAC-M was additionally cured at 60 °C to avoid metastable hydration phases) [10].

## 4. Discussion

The deterioration rate of the different mortar binder types followed the order CAC-M > PC-M > GP-M. The results were obtained via several complementing methods: microhardness (Figure 3b), mass loss (Figure 3a), XRD and TGA-DSC results (Figure 6 and Figure 7), optical and SEM microscopy on degradation depths after 44 days (Figure 5), optical and SEM microscopy on porosity after 22 and 44 days (MIP Table 2), and the external solution pH neutralization rate. All results point out that the deterioration rate in GP-M was significantly less than the other two mortars. According to the estimated degradation depths, the rate of acid attack on GP was at least half of the rate for PC and CAC. Although the deteriorated layer formations in CAC-M (rich in AH_x_ gel) were noticed to be higher, such layers in PC-M were much more porous than for CAC-M and the mechanically intact GP-M. The PC-M performed worse than other two mortars, which is in agreement with the associated chemical alterations. This argument was further supported by the pH results (Figure 2), which showed that the change in pH (i.e., the neutralization rate of the external solution with time) followed the order PC-M > CAC-M > GP-M.

**Figure 8:**
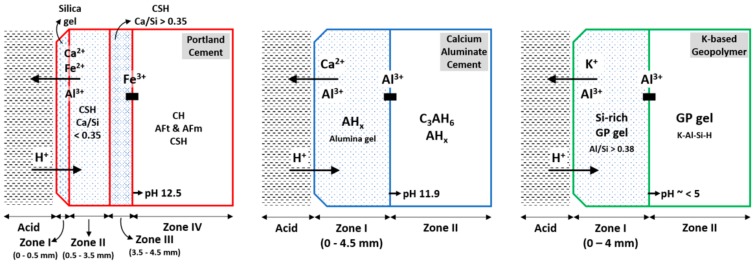
Deterioration process of PC, CAC, and GP-based composites subjected to an acid attack. For PC, zones I and II are classified as deteriorated layers, followed by a transition zone. For CAC, zone I is deteriorated. For GP, zone I is classified as a transition layer, due to the unaffected physical-mechanical properties of geopolymer gel.

Results of the deterioration process of PC, CAC, and GP-based composites are comperatively summerised in Figure 8. The dissolution of portlandite in the PC-M degradative layer was confirmed using XRD (Figure 6), TGA (Figure 7), and SEM-EDS (Figure 5). XRD and TGA results were obtained for all there intervals (0, 22, and 44 days of exposure), which indicated the disappearance of portlandite with an increase in acid exposure time. Gutberlet et al. [2] explained that the acid attack reduced the cementitious matrix’s pH in the pore solution, which subsequently dissolved the portlandite. Further, the acid attack decalcified the remaining phases (AFt, AFm, and C-S-H). This calcium leached zone in PC-M, which was porous (Table 2) and soft (Figure 3b), primarily contained silica-based precipitates and had marginal amounts of brownmillerite (pH 3–4), aluminum hydroxide gel (pH 3–4), and amorphous ferric hydroxide (pH 1–2) [12]. This result was further confirmed using SEM-EDS (Figure 5a), where the Ca/Si ratio was between 0.15 and 0.35. The three major reactions that could possibly occur because on acetic acid attack on a cementitious matrix are given in Equations (1)–(3) [16].

2CH_3_COOH + Ca(OH) _2_ → Ca(CH_3_COO) _2_ + 2H_2_O (1)

6CH_3_COOH + 3CaO^•^Al_2_O_3_^•^6H_2_O (C_3_AH_6_) → 3Ca(CH_3_COO)_2_ + Al_2_O_3_^•^aq + nH_2_O (2)

6CH_3_COOH + 3CaO•2SiO_2_•3H_2_O (C-S-H) → 3 Ca(CH_3_COO)_2_ + 2SiO_2_•aq + nH_2_O (3)

The deterioration process of concrete materials after an acid attack is described by the formation of zones of different mineralogical compositions and pH levels (Figure 8). After such a deterioration gradually moves inward, time advances [16]. Gutberlet et al. [2] characterized two zones: (1) a white, porous, and soft deteriorated layer that consisted of silica gel, in which the pH of pore solution was the same as the external solution, and (2) a transition zone in which the pH of the interstitial solution increased steadily until levelling off at the value for the unaltered core material. The transition zone is a region where the C-H dissolution and C-S-H decalcification undergoes (Figure 8); it also includes a mechanically sound fraction where such hydrates remain intact, as described by Bertron et al. [4].

Scrivener et al. [13,14] reported that CAC could improve the material’s acid neutralization capacity up to 40%, when compared to a PC addition. This result is not in agreement with our results, probably because of our different mix design, which resulted in a higher initial porosity. Apart from CAC’s different chemical composition, the chemistry involved in the diffusion and dissolution–precipitation reactions after an acid attack on the CAC matrix also accounted for such improved resistance (Equation (4)). Herisson et al. [15] suggested that this AH_x_ gel and calcite precipitations (Ca ions and the atmospheric CO_2_ interactions) could fill the pores of the CAC matrix. However, both precipitates remained stable at a pH ranging between 4 and 10, thus dissolving when the pore solution pH decreased further due to the continuous ingress of acid (Equation (5)). 

C_3_AH_6_ + H^+^ → 3Ca^2+^ + 2Al(OH)_x_ (alumina gel) (4)

AH_x_ + H^+^ → 2Al^3+^ (alumina precipitation) + xH_2_O (5)

After 22 days, XRD results (Figure 6b) showed a complete dissolution of the C_3_AH_6_ phase within the degradation layer (and the CaCO_3_ phase; Figure 6). Moreover, Scrivener et al. [17] described that the alumina hydrate (AH_x_) from CAC as stable even at a pH close to 3. Under similar conditions, C_3_AH_6_ decalcifies. The alumina-based precipitates (alumina gel, AH_x_) and fills in the pores of the deteriorated microstructure, which is herein proven with SEM-EDS (Figure 4 and Figure 5) and MIP results (Table 2). Thus, alumina gel limits the free movement of acid species from the external solution. Moreover, CAC is expected to perform better for it lower initial porosities, which can be obtained with a mix design featuring lower water-to-cement ratios (further research is ongoing in this direction). The differences in the acid attack of binder types are schematically illustrated in Figure 8. This is complemented by our SEM-EDS results (Figure 5), which show that both PC and CAC exhibit a sharp jump in calcium profile around a depth of 4 mm, due to the dissolution of Ca(OH)_2_ in PC and C_3_AH_6_ in CAC. Unlike in PC, there is no gradual decrease of Ca toward an outermost surface layer, given that there is no further incongruent decalcification, as was the case for C-S-H in PC. 

For GP-M, the protonation (acid) deterioration is dependent on its pH and can be described as: (i) an ion exchange between the diffusing acid protons (H^+^) and the charge compensating cations (Equation (6), in our case K^+^, but also Na^+^ from a water glass used for GP synthesis) of the geopolymer framework, along with (ii) partial dissolution of the aluminosilicate framework [8,9].

[Si-O-Al-O…]^−^ − K^+^ + H^+^ ↔ [Si-O-Si…] + Al(OH) _4_^−^ + K^+^ (6)

Moreover, the third mechanism (iii) is associated to zeolites crystallization, which causes a decrease in material strength. This result was not corroborated in this study, as it more commonly found in Na-based GPs [16]. A more fundamental thermodynamic solubility description is hampered by a lack of data on GP dissolution and ion-exchange equilibriums [17]. Further, this study is focused on low calcium highly cross-linked polymerized alumina-silicate GPs—i.e., a GP-M system that avoids the Ca-rich dissolvable hydration product formation, which is present in all cement-based materials and alkali-activated Ca-rich binders. Although an acid attack on low calcium alkali-activated binders usually leads to depolymerization and dealumination of the aluminosilicate structure, these binders also show acid corrosion resistance that is superior to PC and CAC conventional binders [18]. Gao et al. [19] recently demonstrated a strong acid attack (0.01 M hydrochloric acid) on a metakaolin-based GP binder for 28 days. Consequently, the alkali ions leached out significantly (exchanged by protons, Equation (6)), but the GP network structure seemed intact when exposed to such an aggressive environment. Results of this study, in particular its SEM-EDS (Figure 5), MIP (Table 2), and microhardness (Figure 3) findings, confirmed that the acid attack altered the GP-Ms microstructure comparatively less than the PC-M and CAC-M cases.

XRD results (Figure 6c) revealed a depolymerization/dealumination of GPs. Further, the results whoed a shift in the amorphous hump, which is in agreement with the dissolution of alumina observed with SEM-EDS (Figure 5c). The acid attack on GPs breaks the Si-O-Al bonds and further leaches out alkalis (Na or K) and Al ions from the binder. This inorganic tetrahedral framework is similar to the zeolite structure, where the solubility depends on the Al/Si ratio, temperature, acid type, and its concentration, etc. [18,19]. The formation of dissolution zones in GP occurs much slower than PC and CAC, where in the transition zone (Figure 8), a binder matrix is composed of silica-rich GP gels (Si rich aluminosilicate) that stay mechanically intact. SEM-EDS elemental profiles (Figure 5) revealed Si dissolution from GP gel until a depth of 2 mm, which is conservatively considered here as a ‘degradation’ zone when compared to the degradation depths of PC and CAC. The transition zone exhibits a gradual dealumination and strong leaching of K^+^ due to an ion exchange [8,9]. Compared to CAC, the slope of the GP dealumination profile is significantly lower, namely 1.0%/mm versus 1.5%/mm, which demonstrates the improved GP resistance of alumina dissolution.

## 5. Conclusions

An accelerated acetic acid attack on the selected mortars resulted in different (deteriorated) layer formations, which surrounded the unaltered core materials that were specific for each binder type: GP, CAC, and PC. The deterioration depths followed the order CAC-M > PC-M > GP-M, where the rate of the acid attack on GP was estimated to be at least half of the rate of attack on PC and CAC. The acid resistance of GP was further noted when classifying the outer zones as ‘transition’, whereas the outer zones for PC and CAC were classified as ‘deteriorated’. The following conclusions were collectively obtained using SEM-EDS, MIP, XRD, and STA results:

- PC binder phases—i.e., CH, C-S-H, AFt, and AFm—are highly soluble for the penetrating acid species; only some C-S-H (Ca/Si < 0.35) remained as an incongruently decalcifying, yet less soluble phase. This results in a highly porous binder matrix that deteriorated microhardness.
- In CAC, a deteriorated layer consists of alumina gel. Although calcium is almost fully depleted, it still results in a lower porosity for the binder matrix than the PC. Due to the dissolution of Ca(OH)_2_ in PC and C_3_AH_6_ in CAC, binders sharply move to the front in the calcium profile, reaching depth of around 4 mm (after 44 days).
- While GP leaches dominate the alkali ions, but the binder aluminosilicate network structure remains stable, dissolving significantly lesser amounts of Si and Al compared to PC and CAC. This results in a less affected porosity and an unaffected microhardness of the binder matrix.

Overall, the findings suggest that the geopolymer-based mortars offer improved acid resistance, which can be a potential alternative to typical cement concretes exposed to various agro-industrial environments.

## Figures and Tables

**Table 1:** Properties of the raw materials that were used in this study.

Raw Material Name	Blaine Value in cm^2^/g	Chemical Composition in wt % (out of 100)								
		CaO	SiO_2_	Fe_2_O_3_	Al_2_O_3_	SO_3_	MgO	K_2_O	Na_2_O	Loss on Ignition
Portland cement	4390	64.6	21.4	4.6	3.7	1.5	0.8	0.4	0.3	2.7
Calcium aluminate cement	3550	40	6	2.5	49.6	0.4	1.5	-	-	-
Metakaolin	26,000	0.1	55	1.4	41	0.05	0.1	0.4	0.05	1.9

**Table 2:** MIP data showing changes in pore structure of the three mortars subjected to the acetic acid attack.

Mortar ID	Time Interval	Amount of (in mm^3^/g)			Total Pore Volume in mm^3^/g	Average Pore Radius in µm
		Gel Pores	Capillary Pores	Macro Pores		
		1–10 nm	0.01–10 µm	>10 µm		
PC-M	*t* + 0	20	35	5	60	0.014
	*t* + 22	9	35	41	85	0.014
	*t* + 44	5	24	82	111	0.034
CAC-M	*t* + 0	1	13	48	62	0.047
	*t* + 22	3	15	68	86	0.038
	*t* + 44	1	8	99	108	0.085
GP-M	*t* + 0	11	16	64	91	0.004
	*t* + 22	39	16	36	92	0.005
	*t* + 44	25	9	57	92	0.008
